# Modernization of Nutritional Assessment in Population Surveys: Integrating Anthropometry, Body Composition, and Biomarkers in the Digital Era

**DOI:** 10.1007/s13668-026-00774-0

**Published:** 2026-05-26

**Authors:** Tatiana Palotta Minari

**Affiliations:** Department of Bioscience, Federal University of São Paulo (UNIFESP), Santos, SP 11015-020 Brazil

**Keywords:** Nutritional assessment, Population surveys, Anthropometry, Body composition, Biochemical indicators, Public health, Digital health, Nutritional surveillance

## Abstract

**Purpose of review:**

This narrative review examines current approaches to nutritional status assessment in population-based surveys, emphasizing the complementary roles of anthropometric measurements, body composition analysis, and biochemical indicators. It aims to critically analyze methodological advances, operational constraints, and emerging strategies to improve the quality and applicability of nutritional surveillance in public health.

**Recent findings:**

Anthropometry remains the most widely used method due to its feasibility and scalability, although its diagnostic capacity is limited. Body composition techniques provide more detailed insights into tissue distribution but are constrained by cost and infrastructure requirements. Biochemical indicators offer high sensitivity for detecting metabolic and micronutrient alterations, yet their use in large-scale surveys is restricted by logistical and ethical challenges. The COVID-19 pandemic exposed vulnerabilities in data collection systems and highlighted the need for more resilient surveillance approaches. In parallel, digital technologies have expanded possibilities for data integration and analysis, although their implementation remains uneven across settings.

**Summary:**

A comprehensive approach to nutritional assessment requires the integration of complementary methods to address the multidimensional nature of malnutrition and chronic disease monitoring. Strengthening population-based surveys depends on balancing methodological rigor with operational feasibility, alongside investments in infrastructure, workforce capacity, and data governance. Advances in digital health may enhance surveillance systems, but their impact will depend on equitable implementation and alignment with public health priorities and equity-oriented strategies.

## Introduction

Assessing nutritional status is a fundamental component of public health, providing essential insights into dietary patterns, health conditions, and epidemiological trends across populations [1–6]. In the context of population-based surveys, nutritional assessment plays a strategic role in guiding public policies, identifying vulnerable groups, and monitoring the distribution and determinants of both undernutrition and non-communicable diseases [2, 7–11].

Comprehensive nutritional assessment encompasses anthropometric, biochemical, clinical, and dietary measures, commonly referred to as the “ABCD” framework [7]. At the population level, however, large-scale nutrition surveillance surveys seldom include full clinical or biochemical assessments due to cost, participant burden, and logistical constraints. Consequently, dietary intake assessment methods, such as 24-hour dietary recalls, food frequency questionnaires (FFQs), and short dietary screeners, are frequently employed to estimate habitual nutrient intakes and identify potential inadequacies [12–15]. While these approaches are practical for large samples, they rely on self-reported intake and are therefore subject to recall bias, misreporting, and errors in portion size estimation. Moreover, nutrient intake estimates depend on food composition databases, which may not fully capture variability in food fortification, reformulation, or branded product differences, potentially leading to under- or overestimation of nutrient adequacy [7, 12–14].

Despite methodological advances, important challenges remain in the implementation of nutritional assessment in large-scale surveys. Operational constraints, including limited infrastructure, variability in professional training, and restricted access to advanced technologies, continue to affect data quality and comparability, particularly in low- and middle-income settings [2, 4–7]. In addition, the growing complexity of nutritional epidemiology—marked by the coexistence of obesity, micronutrient deficiencies, and chronic diseases—demands more integrated and adaptive assessment frameworks [2, 4, 5, 9–18].

In recent years, technological innovations and global health disruptions have further reshaped the field of nutritional surveillance [19–28]. These changes have highlighted both the potential for modernization and the structural limitations of existing systems, reinforcing the need for more resilient and scalable approaches to data collection and analysis [29–40].

This narrative review aims to critically examine current methodologies for assessing nutritional status in population-based surveys, focusing on their applications, limitations, and contributions to public health. By synthesizing evidence across key domains, the study seeks to support the development of more coherent, efficient, and equitable strategies for nutritional surveillance in the contemporary context.

## Methodology

This narrative review was conducted to synthesize current evidence on nutritional status assessment in population-based surveys, with a focus on anthropometry, body composition analysis, and biochemical indicators. A structured literature search was performed across three major scientific databases: SciELO, Web of Science, and PubMed. The search strategy combined controlled descriptors and free-text terms using Boolean operators, including: (“nutritional assessment” OR “nutritional status assessment”) AND (“population surveys” OR “national surveys”) AND (anthropometry OR “body composition” OR “biochemical indicators”) AND (“public health” OR “nutritional epidemiology”) AND (Brazil OR “Brazilian population”) AND (“food and nutrition security” OR “COVID-19” OR “artificial intelligence”). The search was designed to capture studies addressing methodological approaches, epidemiological applications, and policy implications in nutritional assessment.

Eligibility criteria included publications in English or Portuguese between 2002 and 2026 that presented empirical data, methodological discussions, or policy-related analyses relevant to nutritional assessment in population-based contexts. Priority was given to studies conducted in Brazil or in comparable settings, particularly low- and middle-income countries with similar public health system structures and nutritional surveillance challenges, especially those addressing food and nutrition security, and emerging issues such as the COVID-19 pandemic and digital health technologies. Importantly, this study is a narrative review and did not adhere to a formal systematic review protocol (e.g., PRISMA), as its primary objective was to provide an interpretative and critical synthesis of the literature rather than an exhaustive and standardized evidence mapping. A broad range of study designs was considered, including original research articles, systematic reviews, meta-analyses, observational studies, methodological guidelines, and technical reports. Studies addressing methodological innovations, digital health technologies, and emerging approaches in nutritional assessment were also included to provide a broader and up-to-date perspective.

Studies were excluded if they were duplicates, not peer-reviewed, lacked sufficient methodological detail, or were not aligned with the scope of population-based nutritional assessment (e.g., highly specific clinical trials with non-representative samples). Articles focusing primarily or exclusively on dietary intake assessment, without the inclusion of anthropometric, body composition, or biochemical indicators, were excluded, as numerous comprehensive reviews on dietary assessment methodologies are already available, and the present study aimed to focus specifically on anthropometry, body composition, and biochemical indicators in the context of population-based surveys [41–45]. The selection process was guided by relevance, methodological quality, and contribution to the thematic synthesis. A total of 38 publications were included in the final analysis, forming the basis for the narrative integration of evidence presented in this study (Table 1).

**Table 1:** Summary of Scientific Evidence Supporting Nutritional Assessment in Population Surveys

Ref.	Type of Study	Key Findings	Discussion Points	Level of Evidence	Citation
2	Technical manual	Standardized anthropometric data collection	Supports surveillance	High	Brazil. Ministry of Health, [2]
4	National survey (ENANI)	Micronutrient deficiencies in children	Regional disparities	High	Brazil. Ministry of Health/UFRJ, [3]
5	National Health Survey	Health status and nutrition indicators	Epidemiological tool	High	IBGE, [5]
6	Household Budget Survey	Food consumption patterns	Socioeconomic inequalities	High	IBGE, [5]
7	Book chapter	Nutritional assessment fundamentals	Clinical applications	Moderate	Lobo & Cardoso, [7]
10	Narrative review	Tools for metabolic care	Clinical comparisons	Moderate	Taberna et al., [10]
11	Scoping review	Nutritional assessment recommendations	Lack of standardization	Moderate	Soriano-Moreno et al., [11]
12	Narrative review	Nutritional assessment in primary care	Early screening relevance	Moderate	Hurt & McClave, [12]
13	Clinical guide	Quick nutrition assessment in the ICU	Practical use	Moderate	Ferrie, [13]
14	Narrative review	Clinical evaluation and anthropometry	Pediatric relevance	Moderate	Chomtho, [14]
15	Scoping review	Anthropometric formulas	Reference values	Moderate	Bonilla et al., [15]
16	Narrative review	Body composition via radiology	Clinical applications	Moderate	Mazzoccoli, [16]
17	Historical review	Russian body composition studies	Methodological evolution	Moderate	Rudnev & Godina, [17]
18	Technical review	Radiological methods for body composition	Future prospects	Moderate	Linder et al., [18]
19	Narrative review	Body composition in oncology	Clinical relevance	Moderate	Maurya et al., [19]
20	Critical review	Assessment in critically ill patients	Methodological gaps	Moderate	Lambell et al., [20]
21	Narrative review	Ultrasound for body composition	Promising tool	Moderate	Marín Baselga et al., [21]
22	Narrative review	Body composition in sports	New technologies	Moderate	Lukaski & Raymond-Pope, [22]
23	Systematic review + meta-analysis	Body composition and vascular health	Significant associations	High	Nikoohemmat et al., [23]
24	Observational study	BIA linked to COVID-19 severity	Prognostic value	Moderate	Moonen et al., [24]
25	Systematic review	Comparative diagnostic performance of DEXA vs. other radiological modalities	DEXA remains gold standard for osteoporosis detection	High	Wally SF, et al. [25]
26	Imaging-based study	Visceral fat and COVID-19 severity	Clinical relevance	Moderate	Basty et al., [26]
27	Systematic review + meta-analysis	Weight gain during lockdown	Behavioral changes	High	Bakaloudi et al., [27]
28	Narrative review	Eating behaviors during trauma/stress	Stress-diet link	Moderate	Argyrides & Dakanalis, [28]
29	Position Paper	Visceral proteins are unreliable as sole markers of nutritional status	Need for comprehensive assessment beyond albumin and prealbumin	Moderate	Evans et al., [29]
30	Observational Study	Body composition and biochemical markers vary with CKD progression	Importance of individualized nutritional monitoring in CKD	Moderate	Rymarz et al., [30]
31	Review	Criteria for evaluating nutritional biomarkers	Framework for selecting valid, reliable markers in nutrition studies	High	de Vries et al., [31]
32	Narrative Review	Malnutrition in elderly is multifactorial and underdiagnosed	Integration of physical, biochemical, and social indicators	Moderate	Tomasiewicz et al., [32]
33	Cross-sectional Analysis	Food insecurity linked to poor dietary intake and altered biomarkers	Policy implications for child nutrition programs	High	Jun et al., [33]
34	Conceptual Review	Metabolic heterogeneity supports personalized nutrition	Shift from population-based to individualized dietary recommendations	Moderate	Zeisel, [34]
35	Commentary/Perspective	Inflammation affects interpretation of nutritional biomarkers	Need to adjust biomarker interpretation in presence of inflammation	Low to Moderate	Wieringa & Thurnham, [35]
36	Systematic Review	AI, ML, and DL show promise in dietary assessment, prediction, and personalization	Potential for precision nutrition and public health surveillance	High	Theodore Armand et al., [36]
37	Narrative Review	AI applications in food science include nutrient profiling, food safety, and dietary modeling	Integration of AI into food systems and nutrition research	Moderate	Miyazawa et al., [37]
38	Brief Overview	AI supports clinical decision-making in dietetics and nutritional therapy	Use of algorithms for personalized dietary planning	Moderate	Atwal, [38]
39	Narrative Review	AI may transform clinical nutrition through automation and predictive analytics	Future directions in AI-assisted nutritional care	Moderate	Bond et al., [39]
40	Machine learning study	3D body scanner + ML improves obesity classification	Enhances diagnostic precision beyond BMI	High	Jeon, et al., [40]
46	Observational study	Anthropometry, biochemical profile and dietary intake associated with CKD and diabetes	Highlights the importance of integrated nutritional and clinical assessment	Moderate	Ferreira, et al. [46]
47	Narrative review	Seven methods for nutritional status evaluation (ABCDEFG framework)	Provides a comprehensive overview of assessment approaches	Moderate	Zambrano-Villacres, et al. [47]

*ENANI* – National Study on Infant Feeding and Nutrition, *UFRJ* – Federal University of Rio de Janeiro, *IBGE* – Brazilian Institute of Geography and Statistics, *ICU* – Intensive Care Unit, *BIA* – Bioelectrical Impedance Analysis, *CKD* – Chronic Kidney Disease, *AI* – Artificial Intelligence, *ML* – Machine Learning, *DL* – Deep Learning, *DEXA* – Dual-energy X-ray absorptiometry

## Results & Discussion

### Anthropometry

Anthropometry remains the most widely used method for assessing nutritional status in population-based surveys due to its simplicity, low cost, and operational feasibility across diverse settings [2, 5, 7]. It involves the measurement of physical parameters such as body weight, height, and circumferences (e.g., waist, hip, and mid-upper arm), from which indices such as Body Mass Index (BMI) and waist-to-height ratio are derived. These indicators are essential for classifying nutritional status and estimating cardiometabolic risk at the population level [2, 5, 7, 10–15].

In large-scale public health systems, particularly in low- and middle-income countries, anthropometry constitutes the backbone of nutritional surveillance [2, 5, 7]. In Brazil, for example, it is systematically applied in national surveys and routine health information systems, supporting the monitoring of growth, nutritional trends, and health inequalities across different life stages [2, 4–7]. Its scalability allows for broad population coverage, making it indispensable for epidemiological analysis and policy planning.

Despite these advantages, anthropometric assessment presents important limitations. Indicators such as BMI do not differentiate between fat mass and lean mass, potentially leading to misclassification of individuals with atypical body composition profiles. Similarly, circumference-based measures provide only indirect estimates of fat distribution and do not fully capture metabolic risk [11–15]. These constraints limit the ability of anthropometry to detect more complex conditions, such as sarcopenic obesity or early metabolic alterations.

The reliability of anthropometric data is also influenced by operational factors, including evaluator training, adherence to standardized measurement protocols, and equipment calibration. Inconsistent methodologies and measurement errors can compromise data quality and comparability, particularly in large-scale surveys involving multiple teams and regions [12–15].

Recent efforts have focused on improving the quality and consistency of anthropometric measurements through the development of standardized protocols and quality control procedures [11, 12]. International initiatives have emphasized the importance of training, supervision, and methodological harmonization to reduce systematic and random errors in data collection [10–15]. Although technological innovations, such as digital measurement tools and imaging-based approaches, are emerging, their large-scale application remains limited, and traditional anthropometry continues to play a central role in population-based assessments [10, 15].

Overall, anthropometry remains an essential and irreplaceable component of nutritional surveillance. However, its limitations reinforce the need for complementary methods that provide greater specificity and sensitivity in the assessment of body composition and metabolic health.

### Body Composition Analysis

Body composition analysis provides a more detailed evaluation of the distribution of body tissues, including fat mass, lean mass, bone mass, and total body water [16]. This approach allows for a more precise characterization of nutritional status and is particularly relevant for identifying conditions that are not detectable through conventional anthropometric indicators, such as sarcopenia, visceral obesity, and alterations in hydration status [17–23].

A variety of methods are available for assessing body composition, each with distinct levels of accuracy, cost, and feasibility. Bioelectrical impedance analysis (BIA) is widely used due to its non-invasive nature, relatively low cost, and ease of application in both clinical and field settings [19, 20, 24]. However, its accuracy is influenced by factors such as hydration status, recent food intake, and adherence to standardized measurement protocols [24, 25].

Dual-energy X-ray absorptiometry (DEXA) is considered a reference method for body composition assessment, providing precise estimates of fat mass, lean mass, and bone mineral density [18, 26]. Despite its high accuracy, its use in population-based surveys is limited by cost, infrastructure requirements, and the need for specialized personnel. Other techniques, such as air displacement plethysmography, ultrasound, and imaging methods including computed tomography (CT) and magnetic resonance imaging (MRI), offer detailed insights into body composition and fat distribution, particularly at the visceral level [26]. Nevertheless, these approaches are generally restricted to clinical or research settings due to their complexity and limited scalability [21–24].

In recent years, alternative and emerging approaches have been explored to expand access to body composition assessment. These include simplified field methods and digital tools aimed at improving feasibility in large-scale applications. While some studies suggest potential improvements in estimation models, the accuracy and standardization of these methods across diverse populations remain under evaluation. Therefore, caution is warranted when interpreting results derived from novel techniques, particularly in the absence of robust validation studies [21–28].

From a public health perspective, the integration of body composition analysis into large-scale surveys remains limited. Most health systems, especially in resource-constrained settings, rely predominantly on anthropometric indicators due to their operational feasibility. As a result, important aspects of nutritional status, such as fat distribution and muscle mass, are often underexplored in routine surveillance. In public health systems such as the Brazilian Unified Health System (SUS), this gap is even more evident, as advanced technologies are rarely incorporated into routine care due to cost, infrastructure limitations, and the need for specialized personnel [2, 5, 9, 12, 17–19].

Despite these challenges, body composition analysis plays a critical complementary role in nutritional assessment. Its application is particularly valuable in research contexts, clinical settings, and targeted population studies, where greater diagnostic precision is required. Expanding its use in population-based surveys will depend on the development of cost-effective, validated, and scalable methods that can be integrated into existing health systems.

### Biochemical Indicators

Biochemical indicators represent a critical component of nutritional assessment, providing objective and sensitive measures of nutrient status, metabolic function, and physiological responses [10]. Derived from the analysis of biological samples, primarily blood and urine, these markers enable the identification of deficiencies, metabolic imbalances, and inflammatory processes that may not be detectable through anthropometric or body composition methods [10, 28–30].

These indicators are particularly relevant in the context of the global rise in non-communicable diseases, where metabolic alterations often precede clinical manifestations. Biomarkers related to glucose metabolism, lipid profile, micronutrient status, and inflammation contribute to a more precise characterization of nutritional risk and support early detection of conditions such as anemia, hypovitaminosis, insulin resistance, and dyslipidemia [30–32].

Despite their diagnostic value, the application of biochemical indicators in population-based surveys remains limited by significant operational and ethical challenges. Large-scale implementation requires trained personnel, appropriate infrastructure for sample collection and processing, and reliable laboratory capacity. In addition, issues related to sample transport, storage conditions, and quality control can directly affect data validity and comparability across regions [33].

Ethical considerations also play a central role in the use of biochemical data. Procedures such as informed consent, confidentiality, and the management of clinically relevant findings must be carefully addressed, particularly in vulnerable populations. These requirements add complexity to field operations and may limit the feasibility of incorporating biochemical assessments into routine surveillance systems [34].

As a result, biochemical indicators are often restricted to targeted surveys, research initiatives, and specific population groups, rather than being systematically integrated into large-scale monitoring systems. While they offer high specificity and sensitivity, their use in public health must balance analytical precision with logistical feasibility [30–35].

In summary, biochemical assessment provides essential insights into the biological dimension of nutritional status. However, its limitations reinforce the need for its integration with other methodological approaches in order to achieve a comprehensive and operationally viable framework for population-based nutritional surveillance.

### Integrated Nutritional Assessment

Integrated nutritional assessment should not be understood as the simple aggregation of different measurement techniques, but rather as a strategic framework that combines complementary sources of information to enhance the interpretation of population health. By articulating anthropometric, body composition, and biochemical data, it becomes possible to overcome the limitations inherent to each individual method and generate a more accurate and context-sensitive evaluation of nutritional status [2–5, 10].

The added value of this integrated approach lies in its capacity to capture the multidimensional nature of malnutrition and chronic disease monitoring. In contemporary settings, nutritional challenges are increasingly complex, often involving the coexistence of obesity, micronutrient deficiencies, and metabolic disorders within the same population, or even within the same individual. Isolated indicators are frequently insufficient to detect these overlapping conditions, whereas combined approaches allow for more refined identification of risk profiles and health trajectories [12–14].

From a public health perspective, integrated assessment strengthens the analytical power of population-based surveys. It enables the production of more robust and disaggregated data, supporting the identification of priority groups and the design of targeted interventions [2, 4–7]. In national health systems, such as those implemented in Brazil, this approach contributes to the alignment between surveillance, clinical practice, and policy development, enhancing the responsiveness of programs related to food and nutrition security [7–9].

Moreover, integrated data support intersectoral decision-making by linking nutritional outcomes to broader social determinants of health, including income, education, and access to food. This perspective is essential for addressing structural inequalities and for designing policies that go beyond biological risk factors to incorporate social and environmental dimensions [10–14].

However, the implementation of integrated nutritional assessment in large-scale surveys remains challenging. Differences in data availability, methodological heterogeneity, and operational constraints can limit the feasibility of combining multiple indicators in routine practice [2, 5–8]. Ensuring data interoperability, standardization of protocols, and adequate professional training are key requirements for advancing this approach within public health systems.

In practice, this integration is particularly relevant for informing public policies, guiding nutritional programs, and improving the targeting of interventions within primary health care systems. Ultimately, integrated nutritional assessment represents a critical step toward more comprehensive and equitable nutritional surveillance. Its effective application depends not only on methodological advances, but also on the capacity of health systems to incorporate complexity into decision-making and translate data into meaningful public health action [7, 10, 11].

Practical applications of this integrated approach have already been demonstrated in different contexts. For instance, Ferreira et al. [46] demonstrated how the integration of anthropometric measures (waist circumference), body composition indicators (fat mass estimated by bioelectrical impedance analysis), and biochemical markers (lipid profile and glucose metabolism) allowed a more precise identification of diabetes risk among patients with chronic kidney disease, showing that each 1 cm increase in waist circumference raised diabetes risk by 8.4% and each 1 mg/dl increase in VLDL-c raised risk by 8.8% [46]. Similarly, Zambrano-Villacres et al. (2026) proposed the expanded ABCDEFG framework, which articulates anthropometry, biochemistry, clinical, dietary, ecological, functional, and genomic measures to strengthen malnutrition detection and chronic disease monitoring [47]. These examples illustrate how combining anthropometric, body composition, and biochemical data can overcome the limitations of isolated indicators and provide a more comprehensive and context-sensitive evaluation of nutritional health.

### Learnings from the COVID-19 Pandemic Related to Nutritional Assessment

The COVID-19 pandemic significantly affected both the implementation of population-based surveys and the nutritional profile of populations, exposing structural limitations in existing surveillance systems. Public health measures such as social distancing, restrictions on mobility, and the reallocation of healthcare resources disrupted routine data collection, leading to delays, interruptions, or modifications in survey protocols [24, 26].

These operational challenges directly impacted the feasibility of conducting anthropometric measurements, collecting biological samples, and performing in-person assessments. As a result, many surveillance systems experienced gaps in data continuity, reducing the ability to monitor nutritional trends and respond to emerging health needs in a timely manner [24, 26, 27].

Beyond methodological disruptions, the pandemic also altered dietary patterns and nutritional outcomes. Economic instability, job losses, and reduced access to fresh foods contributed to increased food insecurity, particularly among vulnerable populations. At the same time, changes in lifestyle, including reduced physical activity and increased consumption of ultra-processed foods, were associated with rising rates of overweight and obesity, alongside persistent micronutrient deficiencies [27, 28].

The interruption or adaptation of key social and nutrition programs further intensified these effects. Reduced access to school-based feeding initiatives and income transfer programs limited the availability of structured nutritional support, especially for children and low-income households. These changes highlight the interconnected nature of social protection systems and nutritional health, particularly in times of crisis [27, 28].

The pandemic also accelerated the need for more flexible and resilient approaches to nutritional assessment. Alternative strategies, including remote data collection and the use of digital tools, were explored to mitigate disruptions, although their implementation varied widely across settings [27, 28]. These experiences underscored the importance of developing adaptive surveillance systems capable of maintaining data quality under constrained conditions.

In general, the COVID-19 pandemic revealed critical vulnerabilities in nutritional assessment frameworks, while also highlighting opportunities for innovation. Strengthening the resilience, integration, and responsiveness of surveillance systems is essential to ensure continuity of data collection and to support effective public health action in future emergencies.

### Future Perspectives

The modernization of nutritional assessment in population-based surveys is increasingly shaped by advances in digital technologies, offering new possibilities for improving data collection, integration, and analysis. Digital platforms, mobile health applications, and automated data systems have the potential to enhance the efficiency, scalability, and timeliness of nutritional surveillance, particularly in geographically dispersed or resource-constrained settings [36–39].

Among these innovations, the application of artificial intelligence has gained growing attention. Machine learning algorithms and data-driven models can support, but not replace, decision-making in nutritional surveillance, enabling the identification of patterns, prediction of nutritional risks, and optimization of analytical processes. These tools may contribute to more responsive and targeted public health interventions, especially when integrated with existing surveillance systems [30–36].

Recent advances in digital anthropometry have explored the use of three-dimensional body scanners combined with machine learning techniques to improve obesity classification. Jeon et al. (2023) demonstrated that integrating 3D body measurements with algorithmic models enhances the accuracy of obesity diagnosis compared to traditional indices such as BMI, offering a more nuanced understanding of body shape and fat distribution. This approach highlights the potential of artificial intelligence to complement conventional methods, providing scalable and precise tools for nutritional surveillance in population-based contexts [40].

Additional technologies with potential to increase survey accuracy include advanced bioelectrical impedance models, which minimize errors related to hydration status, and mobile digital platforms that enable remote data collection, expanding coverage in hard-to-reach contexts. When combined with innovations such as three-dimensional body scanners and integrated into automated data systems, these tools can enhance comparability and strengthen the predictive capacity of nutritional surveillance [40, 46].

However, the incorporation of digital technologies into nutritional assessment is not without challenges. Issues related to data quality, interoperability, and standardization remain significant barriers. Inconsistent data inputs, variations in measurement protocols, and limited integration between information systems can compromise the reliability and comparability of results. Additionally, disparities in digital infrastructure and access to technology may exacerbate existing health inequalities, particularly in low-resource settings [38, 39]. Beyond these technical barriers, cost-effectiveness and feasibility in real-world survey settings must also be considered. While three-dimensional body scanners and advanced bioelectrical impedance models provide superior accuracy, their high cost and infrastructure requirements limit scalability in low-income or resource-constrained contexts. In contrast, mobile digital platforms represent a more affordable and operationally simple alternative, particularly for remote or underserved populations. Therefore, the adoption of digital technologies should be guided not only by their technical potential but also by pragmatic considerations of cost, simplicity, and equity in access.

Ethical considerations are also central to the digital transformation of nutritional surveillance. The use of large-scale health data requires robust frameworks for data protection, confidentiality, and governance. Ensuring transparency, accountability, and equitable use of data is essential to maintain public trust and to prevent misuse or unintended consequences [38, 39].

Looking ahead, the advancement of nutritional assessment will depend on the balanced integration of technological innovation with structural and institutional capacity. Investments in infrastructure, workforce training, and methodological standardization are necessary to ensure that digital tools are effectively implemented and sustainably maintained. Furthermore, fostering collaboration across sectors, including health, technology, and social policy, will be critical to maximizing the potential of these innovations.

**Fig. 1:**
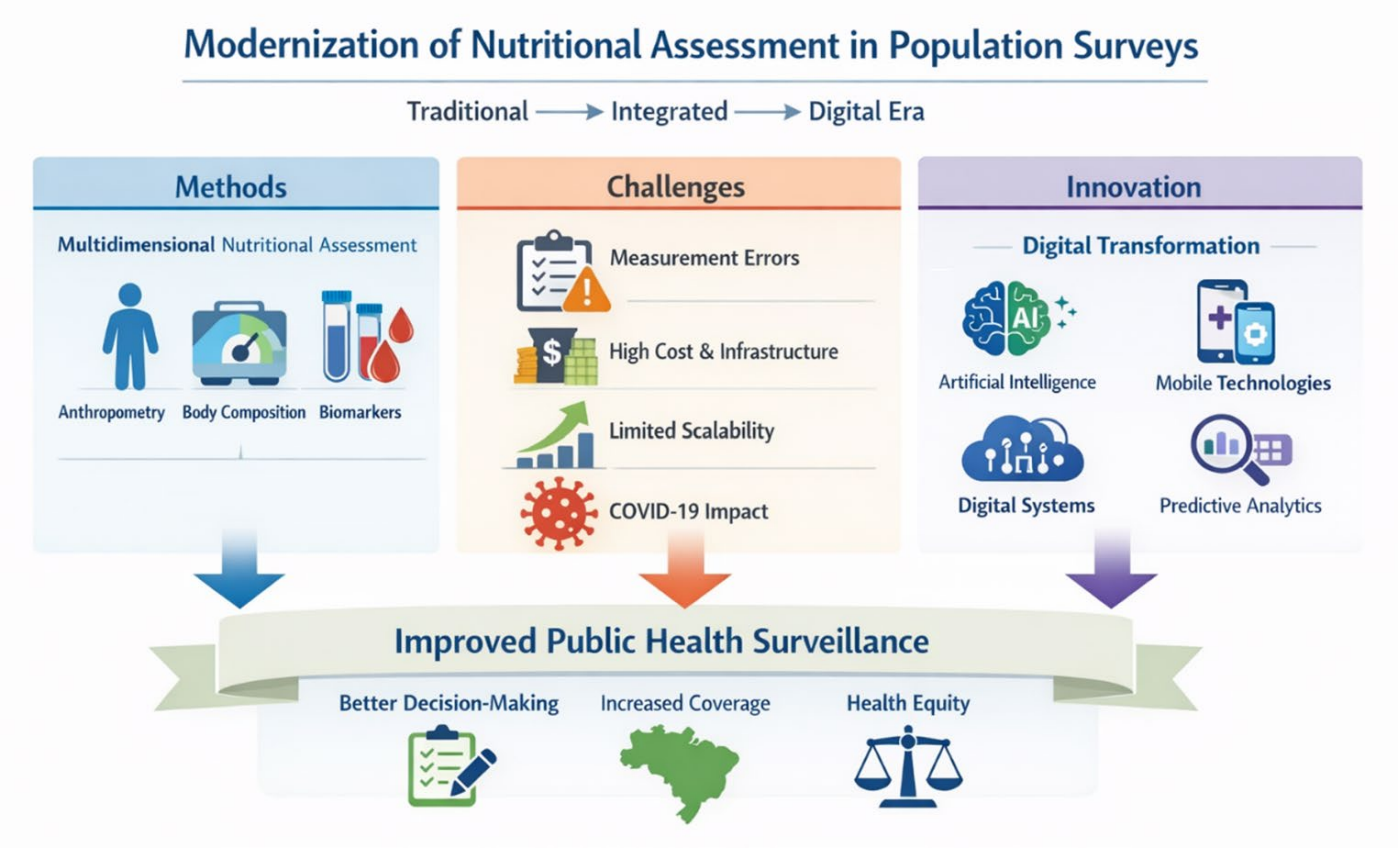
Modernization of nutritional assessment in population surveys. The infographic summarizes the evolution from traditional methods (anthropometry, body composition, and biochemical indicators) toward integrated and digitally enhanced approaches. It highlights operational challenges such as measurement errors, infrastructure costs, limited scalability, and the impact of COVID-19, while also presenting opportunities offered by innovations, including artificial intelligence, mobile technologies, digital systems, and predictive analytics. Arrows converge toward improved public health surveillance, emphasizing outcomes of better decision-making, expanded population coverage, and strengthened health equity

Digital transformation should not be viewed as a replacement for existing methodologies, but as a complementary strategy to enhance their reach and effectiveness. By aligning technological advances with public health priorities, nutritional assessment systems can become more adaptive, inclusive, and capable of addressing the evolving challenges of population health. As nutritional assessment evolves, future strategies must balance methodological rigor with digital innovation. Figure 1 illustrates this trajectory, showing how traditional approaches, structural challenges, and technological advances converge to support more resilient and equitable public health surveillance (Fig. 1).

Despite these contributions, some limitations should be acknowledged. As a narrative review, this study does not follow a formal systematic protocol, which may introduce selection bias and limit the reproducibility of the search and inclusion process. Although efforts were made to include a diverse and relevant body of literature, the synthesis may not capture all available evidence on the topic. In addition, the heterogeneity of study designs, populations, and methodologies across the included publications may affect the comparability and generalizability of the findings. Finally, the rapid evolution of digital technologies in nutrition and public health means that some emerging evidence may not have been fully captured at the time of the review.

## Conclusion

Nutritional assessment in population-based surveys is fundamental for understanding health inequalities and guiding public policy. While anthropometry remains the backbone of surveillance due to its feasibility, complementary methods such as body composition analysis and biochemical indicators provide greater diagnostic precision. Integrating these approaches strengthens the capacity to capture the multidimensional nature of malnutrition and chronic disease monitoring. Persistent challenges, including limited infrastructure, workforce constraints, and lack of methodological standardization, continue to restrict the depth and comparability of data, particularly in resource-limited settings. The COVID-19 pandemic further highlighted these vulnerabilities, reinforcing the need for resilient and adaptable surveillance systems. Emerging digital technologies, including artificial intelligence and automated data platforms, offer promising opportunities to enhance data integration and analytical capacity. Their effective implementation, however, depends on investments in infrastructure, professional training, and ethical governance to ensure equity and reliability. In summary, the modernization of nutritional assessment requires balancing methodological rigor with operational feasibility, while aligning technological innovation with public health priorities. Strengthening surveillance systems through integration, resilience, and equity will be essential to support effective interventions and advance the human right to adequate food.

## Key References

- Lambell KJ, Paris MT, Gonzalez MC, Prado CM. Body Composition Assessment in Critically Ill Adults - Where are We now?. Crit Care Clin. 2025;41(2):283–297. https://doi.org/10.1016/j.ccc.2024.09.006
  - **○ **This review provides a comprehensive update on body composition assessment in critically ill adults, highlighting the clinical relevance of precise measurements for prognosis and therapeutic decision-making in intensive care settings.
- Soriano-Moreno DR, Dolores-Maldonado G, Benites-Bullón A, et al. Recommendations for nutritional assessment across clinical practice guidelines: a scoping review. Clin Nutr ESPEN. 2022;49:201–7. https://doi.org/10.1016/j.clnesp.2022.04.023
  - **○ **This scoping review identifies inconsistencies and gaps in nutritional assessment protocols across clinical guidelines, reinforcing the need for harmonized and evidence-based approaches in public health nutrition.
- Marín Baselga R, Teigell-Muñoz FJ, Porcel JM, Ramos Lázaro J, García Rubio S. Ultrasound for body composition assessment: a narrative review. Intern Emerg Med. 2025;20(1):23–34. https://doi.org/10.1007/s11739-024-03756-8
  - **○ **This article explores the emerging role of ultrasound in body composition analysis, offering a non-invasive and scalable alternative for nutritional assessment in both clinical and field settings
- Nikoohemmat M, Ahmadi AR, Valizadeh A, et al. Association between body composition indices and vascular health: a systematic review and meta-analysis. Eat Weight Disord. 2025;30(1):3. https://doi.org/10.1007/s40519-025-01714-7
  - **○ **This recent meta-analysis synthesizes evidence on the relationship between body composition and vascular health, reinforcing the clinical relevance of precise body composition metrics in predicting cardiovascular risk. It supports the integration of these indices into public health surveillance and preventive strategies.
- Miyazawa T, Miyazawa T, Hiratsuka Y, Toda M, et al. Artificial intelligence in food science and nutrition: a narrative review. Nutr Rev. 2022;80(12):2288–300. https://doi.org/10.1093/nutrit/nuac033
  - **○ **This narrative review explores the emerging applications of artificial intelligence in food science and nutrition, highlighting its potential to enhance dietary analysis, nutritional surveillance, and personalized health interventions. It provides a timely overview of how AI is reshaping methodological approaches in the digital era.

## Data Availability

No datasets were generated or analysed during the current study.

## Declarations

### Patents

This study did not receive any specific patents.

## Abbreviations

Abbreviation: Full Term
AI: Artificial Intelligence
BIA: Bioelectrical Impedance Analysis
BMI: Body Mass Index
BOD POD: Air Displacement Plethysmography
CT: Computed Tomography
DAMs: Digital Anthropometry by Mobile Applications
DEXA: Dual-Energy X-ray Absorptiometry
DL: Deep Learning
FFQs: Food Frequency Questionnaires
MRI: Magnetic Resonance Imaging
ML: Machine Learning
SUS: Unified Health System

## References

[1] Brazil. Ministry of Health. Dietary guidelines for the Brazilian population. 2nd ed. Brasília: Ministry of Health; 2014. Available from: https://bvsms.saude.gov.br/bvs/publicacoes/dietary_guidelines_brazilian_population.pdf. Accessed Jul 2026.

[2] Brazil, Ministry of Health. Food and Nutrition Surveillance System – SISVAN: guidelines for the collection and analysis of anthropometric data in health services. Brasília: Ministry of Health; 2017. Available from: https://bvsms.saude.gov.br/bvs/publicacoes/orientacoes_coleta_analise_dados_antropometricos.pdf. Accessed Jul 2026.

[3] Brazil. Ministry of Health. National food and nutrition policy. Brasília: Ministry of Health; 2011. Available from: https://bvsms.saude.gov.br/bvs/publicacoes/national_food.pdf. Accessed Jul 2026.

[4] Brazil. Ministry of Health. National study on infant feeding and nutrition – ENANI 2019: preliminary results. Rio de Janeiro: Federal University of Rio de Janeiro (UFRJ); 2021. Available from: https://enani.nutricao.ufrj.br/enani-2019/. Accessed Jul 2026.

[5] Brazilian Institute of Geography and Statistics (IBGE). National health survey 2019: perception of health status, lifestyles, chronic diseases, and oral health. Rio de Janeiro: IBGE. 2020. Available from: https://www.ibge.gov.br/en/highlights/29211-the-ibge-will-release-on-november-18-2020-an-additional-volume-of-the-national-survey-of-health-2019-perception-of-health-status-lifestyles-chronic-diseases-and-oral-health.html. Accessed Jul 2026.

[6] Brazilian Institute of Geography and Statistics (IBGE). Household budget survey 2017–2018: analysis of personal food consumption in Brazil. Rio de Janeiro: IBGE. 2020. Available from: https://www.ibge.gov.br/en/statistics/social/population/25610-pof-2017-2018-pof-en.html?edicao=28652. Accessed Jul 2026.

[7] Lobo AS, Cardoso MA. Nutritional status assessment: fundamentals and clinical applications. In: Sichieri R, Coitinho DC, Monteiro CA, editors. Nutrition and public health. Rio de Janeiro: Fiocruz Publishing House; 2002. pp. 121–42. Available from: https://materiais.ead.fiocruz.br/especializacao/alimentacao-e-nutricao-na-atencao-basica/livros-texto/livro_2_web.pdf. Accessed Jul 2026.

[8] Vasconcelos FAG, Batista Filho M. History of the field of food and nutrition in public health in Brazil. Cienc Saude Colet. 2011;16(1):81–90. 10.1590/S1413-81232011000100012. 10.1590/s1413-81232011000100012 21180817

[9] PENSSAN Network – Brazilian Research Network on Food and Nutrition Sovereignty and Security. National survey on food insecurity in the context of the COVID-19 pandemic in Brazil. São Paulo: PENSSAN Network. 2022. Available from: https://basedosdados.org/dataset/6afe59d2-aabe-42a0-b42c-3d6e28952e11?raw_data_source=5b8ceb19-e263-45cc-8ed9-6b125dc3a6ce. Accessed Jul 2026.

[10] Taberna DJ, Navas-Carretero S, Martinez JA. Current nutritional status assessment tools for metabolic care and clinical nutrition. Curr Opin Clin Nutr Metab Care. 2019;22(5):323–8. 10.1097/MCO.0000000000000581. 31246586 10.1097/MCO.0000000000000581

[11] Soriano-Moreno DR, Dolores-Maldonado G, Benites-Bullón A, et al. Recommendations for nutritional assessment across clinical practice guidelines: a scoping review. Clin Nutr ESPEN. 2022;49:201–7. 10.1016/j.clnesp.2022.04.023. 35623814 10.1016/j.clnesp.2022.04.023

[12] Hurt RT, McClave SA. Nutritional assessment in primary care. Med Clin North Am. 2016;100(6):1169–83. 10.1016/j.mcna.2016.06.001. 27745588 10.1016/j.mcna.2016.06.001

[13] Ferrie S. What is nutritional assessment? A quick guide for critical care clinicians. Aust Crit Care. 2020;33(3):295–9. 10.1016/j.aucc.2020.02.005. 32303438 10.1016/j.aucc.2020.02.005

[14] Chomtho S. Clinical evaluation and anthropometry. World Rev Nutr Diet. 2022;124:7–15. 10.1159/000516718. 35240618 10.1159/000516718

[15] Bonilla DA, De León LG, Alexander-Cortez P, et al. Simple anthropometry-based calculations to monitor body composition in athletes: scoping review and reference values. Nutr Health. 2022;28(1):95–109. 10.1177/02601060211002941. 33792415 10.1177/02601060211002941

[16] Mazzoccoli G. Body composition: where and when. Eur J Radiol. 2016;85(8):1456–60. 10.1016/j.ejrad.2015.10.020. 26564096 10.1016/j.ejrad.2015.10.020

[17] Rudnev SG, Godina EZ. Studies on human body composition in Russia: past and present. J Physiol Anthropol. 2022;41(1):18. 10.1186/s40101-022-00291-3. 35505405 10.1186/s40101-022-00291-3 PMC9063054

[18] Linder N, Denecke T, Busse H. Body composition analysis by radiological imaging: methods, applications, and prospects. Rofo. 2024;196(10):1046–54. 10.1055/a-2263-1501. 38569516 10.1055/a-2263-1501

[19] Maurya AK, Aggarwal LM, Choudhary S. Body Composition Analysis Techniques and Its Application in Oncology: A Review. Nutr Cancer. 2024;76(8):666–75. 10.1080/01635581.2024.2353942. 38757446 10.1080/01635581.2024.2353942

[20] Lambell KJ, Paris MT, Gonzalez MC, Prado CM. Body composition assessment in critically ill adults - where are we now? Crit Care Clin. 2025;41(2):283–97. 10.1016/j.ccc.2024.09.006. 40021280 10.1016/j.ccc.2024.09.006

[21] Marín Baselga R, Teigell-Muñoz FJ, Porcel JM, Ramos Lázaro J, García Rubio S. Ultrasound for body composition assessment: a narrative review. Intern Emerg Med. 2025;20(1):23–34. 10.1007/s11739-024-03756-8. 39240412 10.1007/s11739-024-03756-8

[22] Lukaski H, Raymond-Pope CJ. New frontiers of body composition in sport. Int J Sports Med. 2021;42(7):588–601. 10.1055/a-1373-5881. 33621995 10.1055/a-1373-5881 PMC8421000

[23] Nikoohemmat M, Ahmadi AR, Valizadeh A, et al. Association between body composition indices and vascular health: a systematic review and meta-analysis. Eat Weight Disord. 2025;30(1):3. 10.1007/s40519-025-01714-7. 39799535 10.1007/s40519-025-01714-7 PMC11725544

[24] Moonen HPFX, van Zanten FJL, Driessen L, et al. Association of bioelectric impedance analysis body composition and disease severity in COVID-19 hospital ward and ICU patients: the BIAC-19 study. Clin Nutr. 2021;40(4):2328–33. 10.1016/j.clnu.2020.10.023. 33129597 10.1016/j.clnu.2020.10.023 PMC7577288

[25] Wally SF, Wali MF, Hariri OA, et al. Comparative diagnostic performance of dual-energy X-ray absorptiometry and other radiological modalities in osteoporosis detection: a systematic review. J Clin Densitom. 2026;29(1):101637. 10.1016/j.jocd.2025.101637. 41232350 10.1016/j.jocd.2025.101637

[26] Basty N, Sorokin EP, Thanaj M, et al. Abdominal imaging associates body composition with COVID-19 severity. PLoS One. 2023;18(4):e0283506. 10.1371/journal.pone.0283506. 37053189 10.1371/journal.pone.0283506 PMC10101472

[27] Bakaloudi DR, Barazzoni R, Bischoff SC, Breda J, Wickramasinghe K, Chourdakis M. Impact of the first COVID-19 lockdown on body weight: a combined systematic review and a meta-analysis. Clin Nutr. 2022;41(12):3046–54. 10.1016/j.clnu.2021.04.015. 34049749 10.1016/j.clnu.2021.04.015 PMC8056819

[28] Argyrides M, Dakanalis A. Body weight and food/eating-related behaviors during the COVID-19 pandemic or other traumatic or stressful life events. Nutrients. 2024;16(21):3701. 10.3390/nu16213701. 39519536 10.3390/nu16213701 PMC11547810

[29] Evans DC, Corkins MR, Malone A, et al. The use of visceral proteins as nutrition markers: an ASPEN position paper. Nutr Clin Pract. 2021;36(1):22–8. 10.1002/ncp.10588. 33125793 10.1002/ncp.10588

[30] Rymarz A, Zajbt M, Jeznach-Steinhagen A, Woźniak-Kosek A, Niemczyk S. Body composition and biochemical markers of nutrition in non-dialysis-dependent chronic kidney disease patients. Adv Exp Med Biol. 2020;1251:81–9. 10.1007/5584_2019_444. 31745729 10.1007/5584_2019_444

[31] de Vries J, Antoine JM, Burzykowski T, et al. Markers for nutrition studies: review of criteria for the evaluation of markers. Eur J Nutr. 2013;52(7):1685–99. 10.1007/s00394-013-0553-3. 23955424 10.1007/s00394-013-0553-3

[32] Tomasiewicz A, Polański J, Tański W. Advancing the understanding of malnutrition in the elderly population: current insights and future directions. Nutrients. 2024;16(15):2502. 10.3390/nu16152502. 39125381 10.3390/nu16152502 PMC11314143

[33] Jun S, Cowan AE, Dodd KW, et al. Association of food insecurity with dietary intakes and nutritional biomarkers among US children, National Health and Nutrition Examination Survey (NHANES) 2011–2016. Am J Clin Nutr. 2021;114(3):1059–69. 10.1093/ajcn/nqab113. 33964856 10.1093/ajcn/nqab113 PMC8408856

[34] Zeisel SH. Precision (Personalized) Nutrition: Understanding Metabolic Heterogeneity. Annu Rev Food Sci Technol. 2020;11:71–92. 10.1146/annurev-food-032519-051736. 31928426 10.1146/annurev-food-032519-051736

[35] Wieringa FT, Thurnham DI. Inflammation, biomarkers, and real nutritional status. J Nutr. 2023;153(3):605–7. 10.1016/j.tjnut.2023.01.007. 36931742 10.1016/j.tjnut.2023.01.007

[36] Theodore Armand TP, Nfor KA, Kim JI, Kim HC. Applications of artificial intelligence, machine learning, and deep learning in nutrition: A systematic review. Nutrients. 2024;16(7):1073. 10.3390/nu16071073. 38613106 10.3390/nu16071073 PMC11013624

[37] Miyazawa T, Hiratsuka Y, Toda M, et al. Artificial intelligence in food science and nutrition: A narrative review. Nutr Rev. 2022;80(12):2288–300. 10.1093/nutrit/nuac033. 35640275 10.1093/nutrit/nuac033

[38] Atwal K. Artificial intelligence in clinical nutrition and dietetics: A brief overview of current evidence. Nutr Clin Pract. 2024;39(4):736–42. 10.1002/ncp.11150. 38591653 10.1002/ncp.11150

[39] Bond A, Mccay K, Lal S. Artificial intelligence & clinical nutrition: What the future might have in store. Clin Nutr ESPEN. 2023;57:542–9. 10.1016/j.clnesp.2023.07.082. 37739704 10.1016/j.clnesp.2023.07.082

[40] Jeon S, Kim M, Yoon J, Lee S, Youm S. Machine learning-based obesity classification considering 3D body scanner measurements. Sci Rep. 2023;13(1):3299. 10.1038/s41598-023-30434-0. 36843097 10.1038/s41598-023-30434-0 PMC9968712

[41] Dao MC, Subar AF, Warthon-Medina M, et al. Dietary assessment toolkits: an overview. Public Health Nutr. 2019;22(3):404–18. 10.1017/S1368980018002951. 30428939 10.1017/S1368980018002951 PMC6368251

[42] O’Hara C, Gibney ER. Dietary intake assessment using a novel, generic meal-based recall and a 24-hour recall: comparison study. J Med Internet Res. 2024;26:e48817. 10.2196/48817. 38354039 10.2196/48817 PMC10902769

[43] Baygi F, Mohammadi-Nasrabadi F, Zyriax BC, et al. Global overview of dietary outcomes and dietary intake assessment methods in maritime settings: a systematic review. *BMC Public Health*. 2021;21(1):1579. 10.1186/s12889-021-11593-z. 34419000 10.1186/s12889-021-11593-z PMC8379789

[44] Murai U, Tajima R, Matsumoto M, et al. Validation of dietary intake estimated by web-based dietary assessment methods and usability using dietary records or 24-h dietary recalls: a scoping review. Nutrients. 2023;15(8):1816. 10.3390/nu15081816. 37111035 10.3390/nu15081816 PMC10141001

[45] Matsumoto M, Murakami K, Yuan X, et al. A scoping review of dietary assessment questionnaires potentially suitable for assessing habitual dietary intake in the National Health and Nutrition Survey, Japan. J Nutr Sci. 2024;13:e8. 10.1017/jns.2024.1. 38379590 10.1017/jns.2024.1 PMC10877143

[46] Ferreira ES, da Silva LS, da Costa GD, Moreira TR, Borges LD, Cotta RMM. Dietary intake, anthropometric measurements, biochemistry profile and their associations with chronic kidney disease and diabetes mellitus. J Nutr Sci. 2020;9:e45. 10.1017/jns.2020.38. 33101662 10.1017/jns.2020.38 PMC7550961

[47] Zambrano-Villacres R, Arteaga-Pazmiño C, Guevara Castillo WD, et al. The seven methods for the evaluation of nutritional status—ABCDEFG: narrative review. Appl Sci. 2026;16(2):845. 10.3390/app16020845.

